# Treatment options and their uptake among women with symptoms of perinatal depression: exploratory study in Norway and Portugal

**DOI:** 10.1192/bjo.2023.56

**Published:** 2023-05-04

**Authors:** Ana Fonseca, Mariana Branquinho, Fabiana Monteiro, Anabela Araújo-Pedrosa, Ludvig D. Bjørndal, Angela Lupattelli

**Affiliations:** Center for Research in Neuropsychology and Cognitive Behavioral Intervention, Faculty of Psychology and Educational Sciences, University of Coimbra, Portugal; Center for Research in Neuropsychology and Cognitive Behavioral Intervention, Faculty of Psychology and Educational Sciences, University of Coimbra, Portugal; and Clinical Psychology Service, Department of Gynaecology, Obstetrics, Reproduction and Neonatology (Maternity Daniel de Matos), Centro Hospitalar e Universitário de Coimbra, Portugal; PROMENTA Research Center, Department of Psychology, University of Oslo, Norway; Pharmacoepidemiology and Drug Safety Research Group, Department of Pharmacy, Faculty of Mathematics and Natural Sciences, University of Oslo, Norway; University of Coimbra, Center for Research in Neuropsychology and Cognitive Behavioral Intervention, Faculty of Psychology and Educational Sciences, Portugal

**Keywords:** Perinatal period, depressive symptoms, treatment options, help-seeking, exploratory study

## Abstract

**Background:**

Perinatal depression is the most undertreated clinical condition during the perinatal period. Knowledge about women's decision-making in seeking and receiving treatment is scarce.

**Aims:**

To investigate and compare treatment option uptake in perinatal women with depressive symptoms in Portugal and Norway, and to identify sociodemographic and health-related factors associated with treatment uptake.

**Method:**

Participants were women resident in Portugal or Norway (≥18 years) who were pregnant or had given birth in the past 12 months, who presented with active depressive symptoms (Edinburgh Postnatal Depression Scale score ≥10). In an electronic questionnaire, women reported treatment received and sociodemographic and health-related factors.

**Results:**

The sample included 416 women from Portugal and 169 from Norway, of which 79.8% and 53.9%, respectively, were not receiving any treatment. Most Portuguese women were receiving psychological treatment, either alone (45.2%) or combined with pharmacological treatment (21.4%). Most Norwegian participants were receiving only pharmacological (36.5%) or combined treatment (35.4%). Compared with the Portuguese sample, a higher proportion of Norwegian women started treatment before pregnancy (*P* < 0.001). In Portugal, lower depressive symptoms and self-reported psychopathology were significantly associated with higher likelihood of receiving treatment.

**Conclusions:**

We found that, in both Norway and Portugal, a substantial number of perinatal women with depressive symptoms do not receive any treatment. Differences exist regarding the chosen treatment option and timing of treatment initiation in the two countries. Only mental health-related factors were associated with treatment uptake for perinatal depression in Portugal. Our results highlight the importance of implementing strategies aimed to improve help-seeking behaviours.

Pregnancy and postpartum are periods of greater vulnerability for the development of perinatal depression,^1^ which is a prevalent condition worldwide (pooled prevalence of 11.9%).^2^ Perinatal depression has detrimental consequences not only for the mother's health, but also for the infant's development,^3^ with high socioeconomical costs.^4^

## Underdiagnosed and undertreated

Despite the high prevalence and negative consequences of perinatal depression, it remains the most underdiagnosed and undertreated mental health condition during the perinatal period.^5,6^ Effective treatment options are available, which include psychotherapy, pharmacological treatment with antidepressants or a combination thereof,^7,8^ and the decision-making process to select the best option for each case may be complex. Evidence suggests that perinatal women prefer non-medication-based treatments for depression, such as psychotherapy,^9^ or combined treatment (medication and psychotherapy),^10,11^ when compared with medication alone. This may be partly attributable to concerns regarding the potential teratogenic effects of medication,^9,12^ which could reflect the discordant findings and lack of information concerning the use of psychotropics and their effects on neonatal and maternal safety.

It can be challenging for clinicians and women to assess the risk of pharmacotherapy versus the risk of not treating maternal illness.^13,14^ Moreover, a significant proportion of women do not seek professional help for mental health problems during the perinatal period.^15,16^ This complex scenario, coupled with the limited availability of perinatal-specific clinical practice guidelines across Europe,^17^ often leaves women and healthcare professionals with a treatment dilemma.^1^

## Two countries

The present study focuses on two countries, Portugal and Norway, with a birth rate of 8.20/1000 inhabitants and 9.80/1000 inhabitants, respectively, and similar estimates of perinatal depression.^18,19^ In both countries, there is no universal screening for perinatal depression, but women have access to free maternity care. The lack of specialised perinatal mental health services, referral pathways and standardised screening procedures in the two countries may hinder efficient access to treatment for women's perinatal mental health disorders.^20,21^ Although in Norway, there are specific clinical practice guidelines for perinatal depression management, there are no such guidelines in Portugal.^17^ This latter difference could possibly influence how women with perinatal depressive symptoms are treated. Comparing Norway and Portugal in terms of treatment uptake for depressive symptoms could therefore yield insights into important similarities and differences between the countries regarding treatment for depressive symptoms in the perinatal period.

The present exploratory study aimed (a) to map and compare how women with active symptoms of depression were treated in the perinatal period in Portugal and Norway, overall and by treatment options; and (b) to identify maternal sociodemographic and health-related factors, including perinatal status, associated with treatment uptake in the two countries.

## Method

### Study design and participants

In Norway, participants were recruited from the HEALTHx2 study. The HEALTHx2 study is a cross-sectional, sequential mixed-methods study, in which data was collected from all regions of Norway between June 2020 and June 2021. The quantitative component preceded the qualitative one. The current study used solely quantitative cross-sectional data, which were collected using an electronic questionnaire administered via ‘Nettskjema’, provided by the University of Oslo. Participants could choose to access the questionnaire anonymously or by using their national identification number. Information about the study was posted in multiple pregnancy and motherhood-related websites and apps, on social media and in brochures, which were distributed at various psychiatric policlinics, hospital psychiatric departments and maternity health clinics. The recruitment sites and complete questionnaire are described in Supplementary Files 1 and 2 available at https://doi.org/10.1192/bjo.2023.56. Women were eligible to participate if they were aged 18–55 years; pregnant or had given birth within the past 5 years; and had a mental illness and had been offered antidepressant treatment in the past 5 years. For this specific study, we included only pregnant women and those who gave birth in the past year who reported active depressive symptoms (a score of ≥10 on the Edinburgh Postnatal Depression Scale; EPDS^22^) at the time of questionnaire completion.

In Portugal, participants were recruited from the Women Choose Health study, a cross-sectional survey advertised between February 2021 and February 2022. Data was collected using an electronic questionnaire on the Limesurvey platform hosted at the Faculty of Psychology and Educational Sciences of the University of Coimbra. The study was advertised through social networks (e.g. Facebook/Instagram), through unpaid and cross-paid posting campaigns targeting women of reproductive age (18–45 years) with interest in maternity topics. Parenting-related forums were also used for study advertisement. The Women Choose Health study was advertised to all women in the perinatal period, as a study concerning decision-making processes about treatment options for mental health problems during the perinatal period (i.e. participation in the study did not require women to have been offered antidepressants or have a diagnosed mental illness). Inclusion criteria for study participation were being an adult woman (≥18 years) who was pregnant or had given birth in the past 12 months. For the purpose of the current study, women were included they presented active symptoms of depression at the time of questionnaire completion (a score of ≥10 on the EPDS^22^).

**Fig. 1:**
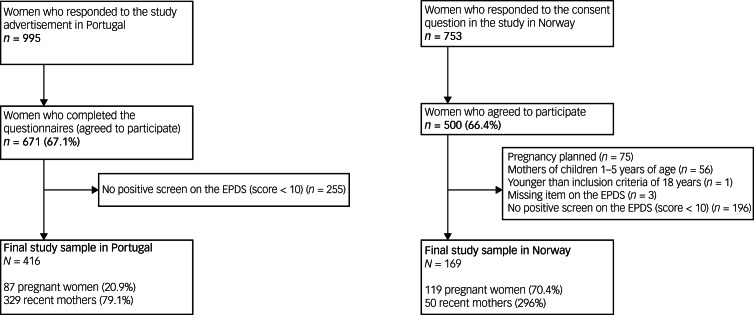
Data flow to achieve the final study sample in Portugal and Norway. EPDS, Edinburgh Perinatal Depression Scale.

The data flow to achieve the final study sample both in Portugal and Norway is presented in Fig. 1. The questionnaire was first developed in Norwegian and English, and most of its sections were then translated into Portuguese (see Supplementary File 3).

The authors assert that all procedures contributing to this work comply with the ethical standards of the relevant national and institutional committees on human experimentation and with the Helsinki Declaration of 1975, as revised in 2008. Electronic informed consent was given by each participant in Norway and Portugal. The Regional Ethics Committee in Norway, region Southeast (reference number 94347), and the Norwegian Centre for Research Data (reference number 943055) approved the study in Norway. The Ethics Committee of the Faculty of Psychology – University of Coimbra approved the study in Portugal (CEDI_13012021). Written informed consent was obtained from all participants.

### Measures

#### Depressive symptoms

Active depressive symptoms were measured with the EPDS,^22^ a self-report ten-item scale, which has been validated in pregnancy and postpartum for major and minor depression in clinical settings both in Norway and Portugal, and has good psychometric properties.^23,24^ Women were asked to rate whether each item reflected how they had been feeling in the past 7 days. Each item response was scored from 0 to 3 on an ordinal scale, producing a total EPDS score with a range of 0–30. Higher scores indicate worse symptoms, with a score of ≥10 being indicative of active depressive symptoms.

#### Mental health factors

Previous history and current mental health problems were measured via self-report items in which participants could indicate the mental illness they currently or previously had within a predefined list, including: depression, anxiety, obsessive–compulsive disorders, eating disorders, other mental illness and no mental illness. Participants were also asked to indicate the time points at which they had a mental illness, from before pregnancy (>1 year or <1 year before pregnancy) through pregnancy (trimesters 1–3), and up to 1 year after birth (0–6 and 7–12 months postpartum). Based on the time periods checked by the women, we ascertained whether the mental illness began before, during or after pregnancy. To measure women's mental health burden, we counted the number of different illnesses reported across the available time periods.

Perceived stigma related to mental illness was measured with the Indifference to Stigma subscale from the Attitudes Toward Seeking Professional Psychological Help Scale (ATSPPHS).^25^ This subscale comprises eight items and we selected the four items with the highest factor loadings on the perceived stigma factor, as shown by Mackenzie et al.^25^ These are: ‘Having been mentally ill carries with it a burden of shame’, ‘I would be embarrassed if my neighbour saw me going into the office of a professional who deals with psychological problems’, ‘Important people in my life would think less of me if they were to find out that I was experiencing psychological problems’ and ‘Having been diagnosed with a mental disorder is a blot on a person's life’. Participants could indicate the extent to which they agreed or disagreed on each item, with a score ranging from 0 to 4. Scores across items were averaged and a higher score corresponded to greater indifference to stigma (i.e. more positive attitudes). This was modelled as a numeric variable. The ATSPPHS has a Portuguese version, which has shown good psychometric properties.^26^ For data collection in Norway, the scale was translated into Norwegian and back-translated by two independent translators.

#### Treatment options

Women were asked if they had previously received or were currently receiving psychological therapy or pharmacological therapy (dichotomised as yes/no for each option) and, if yes, the type of therapy and when they received it. Type of psychological therapy was grouped into individual and/or group therapy. Concerning pharmacological therapy, women were presented with a list of all antidepressants marketed in the countries, and were asked to report which substance they used and the timing of use. To enhance recall, examples of brand names of the various antidepressants were also presented. For both psychotherapy and antidepressants, the time options for their use/receipt were ‘before pregnancy’, ‘during pregnancy’ and ‘during postpartum’. Based on the type of treatment used at the time of questionnaire completion, women were classified as receiving only psychological treatment, only psychopharmacological with antidepressants, or a combination of both.

#### Sociodemographic and lifestyle characteristics

These included women's age, number of prior children, marital status, gestational week for pregnant women or months since childbirth for recent mothers at the time of questionnaire completion, educational attainment and work situation at the time of questionnaire conception, and whether the current or latest pregnancy was planned. The questions were based on a prior web-based, cross-sectional study conducted among pregnant women in Norway.^27^ To avoid data sparsity, maternal variables were categorised as shown in Table 1.

**Table 1:** Sociodemographic, pregnancy and lifestyle characteristics of participants by treatment status for both Portuguese and Norwegian samples

	Portuguese sample (*N* = 416)			Norwegian sample (*N* = 169)		
	On any treatment (*n* = 84)	No treatment (*n* = 332)	Total (*N* = 416)	On any treatment (*n* = 96)	No treatment (*n* = 73)	Total (*N* = 169)
	*n* (%)	*n* (%)	*n* (%)	*n* (%)	*n* (%)	*n* (%)
Sociodemographic characteristics						
Age, years, mean (s.d.)	34.3 (4.9)	34.7 (4.7)	34.6 (4.7)	30.8 (4.4)	30.2 (5.1)	30.6 (4.8)
Marital status						
Married/living together	78 (92.9)	308 (92.8)	386 (92.8)	91 (94.8)	71 (97.3)	162 (95.9)
Single/divorced	6 (7.1)	24 (7.2)	30 (7.2)	5 (5.2)	<5	7 (4.1)
Educational level						
Less than high school	4 (4.8)	12 (3.6)	16 (3.8)	<5	8 (11.0)	12 (7.1)
High school	12 (14.3)	71 (21.4)	83 (20.0)	25 (26.0)	17 (23.3)	42 (24.9)
University	68 (81.0)	249 (75.0)	317 (76.2)	67 (69.8)	48 (65.8)	115 (68.1)
Professional status						
Occupied	72 (85.7%)	306 (93.3)	378 (91.7)	67 (69.8)	55 (75.3)	122 (72.2)
Unemployed	8 (9.5%)	17 (5.2)	25 (6.1)	11 (11.5)	8 (11.1)	19 (11.2)
Others	4 (4.8%)	5 (1.5)	9 (2.2)	18 (18.8)	10 (13.7)	28 (16.6)
Number of children*						
0	17 (20.2)	29 (8.7)*	46 (11.1)	56 (58.3)	52 (71.2)	108 (63.9)
1	47 (56.0)	197 (59.3)	244 (58.7)	30 (31.3)	16 (21.9)	46 (27.2)
2	16 (19.0)	90 (27.1)	106 (25.5)	10 (10.4)	<5	13 (7.7)
More than 2	4 (4.8)	16 (4.8)	20 (4.8)	−	<5	<5
Perinatal status and pregnancy characteristics						
Perinatal period						
Pregnancy	31 (36.9)	56 (16.9)*	87 (20.9)	70 (72.9)	49 (67.1)	119 (70.4)
Postpartum	53 (63.1)	276 (83.1)	329 (79.1)	26 (27.1)	24 (32.9)	50 (29.6)
Trimester of pregnancy^a^						
First	3 (9.7)	2 (5.4)	6 (6.9)	20 (28.6)	22 (44.9)	42 (35.3)
Second	12 (38.7)	21 (37.5)	33 (37.9)	37 (52.9)	17 (34.7)	54 (45.4)
Third	16 (51.6)	32 (57.1)	48 (55.2)	13 (18.6)	10 (20.4)	23 (19.3)
Postpartum period (months)^b^						
Less than 1 month	2 (3.8)	24 (8.7)	26 (7.9)	−	−	−
1–3 months	26 (49.1)	113 (40.9)	139 (42.2)	9 (34.6)	7 (34.6)	16 (32.0)
4–6 months	14 (26.4)	65 (23.6)	79 (24.0)	8 (30.8)	10 (41.7)	18 (36.0)
7–9 months	6 (11.3)	39 (14.1)	45 (13.7)	5 (23.1)	5 (20.8)	11 (22.0)
10–12 months	5 (9.4)	35 (12.7)	40 (12.2)	<5	<5	<5
Pregnancy planning						
Planned	52 (61.9)	237 (71.4)	289 (69.5)	61 (63.5)	50 (68.5)	111 (66.1)
Unplanned	13 (15.5)	36 (10.8)	49 (11.8)	25 (26.0)	7 (0.6)	16 (9.5)
Unplanned, but not completely unexpected	19 (22.6)	59 (17.8)	78 (18.8)	9 (9.4)	16 (21.9)	41 (24.4)
Lifestyle factors after awareness of pregnancy						
Alcohol consumption during pregnancy						
Yes	7 (8.3)	31 (9.3)	38 (9.1)	<5	<5	6 (3.6)
No	76 (90.5)	296 (89.2)	372 (89.4)	93 (96.9)	70 (95.9)	163 (96.5)
Don't remember	1 (1.2)	5 (1.5)	6 (1.4)	−	−	−
Smoke consumption during pregnancy						
Yes, daily	6 (7.1)	25 (7.5)	31 (7.5)	<5	−	<5
Yes, sometimes (two/three times per week)	5 (6.0)	18 (5.4)	23 (5.5)	8 (8.3)	<5	10 (5.9)
No	73 (86.9)	289 (87.0)	362 (87.0)	87 (90.6)	71 (97.3)	158 (93.5)

a. Only for pregnant women.

b. Only for women in the postpartum period. Numbers may not add up to total because of missing values; missing values on pregnancy planning for one woman in Norway.

* *P* < 0.05 for the comparison of the distribution of maternal characteristics by treatment status groups in each sample.

### Statistical analysis

The analysis was conducted separately in the two samples. Descriptive statistics were first conducted. The difference in the distribution of maternal characteristics by treatment status was tested with *χ*^2^-tests, and *t*-tests were used for numeric variables. Clinical characteristics by treatment status were additionally stratified by perinatal status (pregnant or postpartum) at the time of study participation. The proportions of treatment type uptake and its correlates were compared for the samples in Portugal and Norway, using MedCalc version 20.218 for Windows (MedCalc Software Ltd, Ostend, Belgium; https://www.medcalc.org/); this produced the absolute difference in proportions between the two samples, with the corresponding 95% confidence interval.

To determine which maternal factors were related to treatment uptake during the perinatal period, we conducted multiple logistic regression models (separately for the Portuguese and Norwegian samples) comparing women receiving treatment with women receiving no treatment. These models were built following the purposeful selection approach.^28^ Candidate variables were selected based on *P* < 0.20 in a univariable logistic regression model; variables having no role (*P* > 0.05) or yielding a change smaller than 15% in the *β*-coefficients of the retained variables were removed. We examined the effect of a vast array of mental health and sociodemographic factors on treatment uptake. Candidate mental health variables included EPDS numeric score, number of self-reported mental illnesses and perceived stigma related to mental illness. Candidate demographic variables comprised age, marital status, work situation, education, perinatal status and planned pregnancy. Missing data were only present for the variable pregnancy planning (<0.6% missing). The final multiple regression models included statistically significant factors (for each country) and clinically relevant factors (i.e. age, education). Results are presented as odds ratio with the corresponding 95% confidence intervals. All statistical analyses were conducted with Stata MP version 17 for Windows in Norway and SPSS version 22 for Windows in Portugal.

## Results

### Characteristics of the Portuguese and Norwegian samples

In Norway, 196 women with no positive screen on the EPDS were excluded, and 255 Portuguese women were excluded for the same reason. The final sample included 416 women in Portugal and 169 in Norway with active symptoms of depression in the perinatal period.

The majority of women in Norway were pregnant (119/169, 70.4%), whereas most women in Portugal were recent mothers (329/416, 79.0%). There were 84 (20.2%) and 78 (46.2%) women who were currently receiving treatment in Portugal and Norway, respectively, whereas 332 (79.8%) and 91 (53.9%) women were not receiving any treatment. Table 1 presents the sociodemographic and health characteristics of women in both samples, overall and by treatment status.

The mean age of women was 34.6 years in the Portuguese sample and 30.6 years in the Norwegian sample. In both samples, most women had a university education and were married/cohabiting. In the Portuguese sample, a higher proportion of women receiving treatment had no prior children and were currently pregnant, compared with those receiving no treatment. No significant differences between groups were found in the Norwegian sample.

Table 2 presents the clinical characteristics of women in both samples, overall and by treatment status, as well as separately for pregnant and postpartum women. Most women in the Portuguese sample reported only one mental illness, whereas psychiatric comorbidity was common in Norway. For women in Norway, both depression and anxiety were most often pre-existing from before pregnancy; in Portugal, these disorders started across all time periods, including pregnancy. The overall mean EPDS score was similar in the two countries (14.1 in Portugal, 14.0 in Norway). Women not receiving any treatment in Portugal reported significantly higher EPDS scores than women receiving treatment, and this was also found for pregnant and postpartum women when analysed separately. The EPDS score was similar for both treatment groups in Norway.

**Table 2:** Clinical characteristics of participants by treatment status for both Portuguese and Norwegian samples

	Portuguese sample (*n* = 416)			Norwegian sample (*n* = 169)		
	On any treatment (*n* = 84)	No treatment (*n* = 332)	Total (*N* = 78)	On any treatment (*n* = 96)	No treatment (*n* = 73)	Total (*N* = 169)
	*n* (%)	*n* (%)	*n* (%)	*n* (%)	*n* (%)	*n* (%)
Self-reported number of mental illness						
None	−	192 (57.8)	192 (46.2)	−	<5	<5
1	38 (45.2)	80 (24.1)	118 (28.4)	13 (13.5)	16 (21.9)	29 (17.2)
2	33 (39.3)	51 (15.4)	84 (20.2)	39 (40.6)	25 (34.3)	64 (37.9)
3 or more	13 (15.5)	9 (2.7)	22 (5.3)	44 (45.8)	31 (42.5)	75 (44.4)
Beginning of self-reported depression^a^						
Before pregnancy	32 (52.5)	50 (57.5)*	82 (55.4)	81 (84.4)	57 (78.1)	138 (81.7)
Pregnancy	13 (21.3)	6 (6.9)	19 (12.8)	<5	<5	8 (4.7)
Postpartum	16 (26.2)	31 (35.6)	47 (31.8)	<5	<5	<5
Beginning of self-reported anxiety^b^						
Before pregnancy	23 (37.1)	35 (34.0)	58 (35.2)	70 (72.9)	55 (75.3)	125 (74.0)
Pregnancy	19 (30.6)	31 (30.1)	50 (30.3)	9 (9.4)	<5	11 (6.5)
Postpartum	20 (32.3)	37 (35.9)	57 (34.5)	<5	−	<5
EPDS score, mean (s.d.)	12.8 (5.1)	14.4 (3.8)*	14.1 (4.2)	14.1 (3.7)	13.8 (3.2)	14.0 (3.8)
Perceived stigma for mental illness, mean (s.d.)	3.2 (0.9)	3.0 (1.0)	3.1 (1.0)	2.1 (1.0)	2.3 (1.1)	2.2 (1.1)

	Pregnant women (*n* = 87)			Pregnant women (*n* = 119)		
	On any treatment (*n* = 31)	No treatment (*n* = 56)	Total (*N* = 87)	On any treatment (*n* = 70)	No treatment (*n* = 49)	Total (*N* = 119)
*n* (%)	*n* (%)	*n* (%)	*n* (%)	*n* (%)	*n* (%)
Self-reported number of mental illness						
None	−	19 (33.9)	19 (21.8)	−	<5	<5
1	16 (51.6)	21 (37.5)	37 (42.5)	15 (21.4)	13 (26.5)	58 (23.5)
2	10 (32.3)	14 (25.0)	24 (27.6)	33 (47.1)	25 (51.0)	58 (48.7)
3 or more	5 (16.1)	2 (3.6)	7 (8.0)	22 (31.4)	10 (20.4)	32 (26.9)
Beginning of self-reported depression^a^						
Before pregnancy	12 (38.7)	11 (19.6)	23 (26.4)	60 (85.7)	37 (75.5)	97 (81.5)
Pregnancy	9 (29.0)	6 (10.7)	15 (17.2)	<5	<5	5 (4.2)
Postpartum	−	−	−	−	−	−
Beginning of self-reported anxiety^b^						
Before pregnancy	10 (32.3)	10 (17.9)	20 (23.0)	53 (75.7)	37 (75.5)	90 (75.6)
Pregnancy	13 (41.9)	17 (30.4)	30 (34.5)	6 (8.6)	<5	7 (5.9)
Postpartum	−	−	−	−	−	−
EPDS score, mean (s.d.)	12.3 (4.9)	14.8 (4.4)*	13.9 (4.7)	13.8 (3.4)	14.0 (3.4)	13.9 (3.4)
Perceived stigma for mental illness, mean (s.d.)	3.1 (0.9)	3.1 (1.1)	3.1 (1.1)	2.10 (1.0)	2.4 (1.1)	2.2 (1.1)

	Postpartum women (*n* = 329)			Postpartum women (*n* = 50)		
	On any treatment (*n* = 53)	No treatment (*n* = 276)	Total (*N* = 329)	On any treatment (*n* = 26)	No treatment (*n* = 24)	Total (*N* = 50)
	*n* (%)	*n* (%)	*n* (%)	*n* (%)	*n* (%)	*n* (%)
Self-reported number of mental illness						
None	−	173 (62.7)	173 (52.6)	−	−	−
1	22 (41.5)	59 (21.4)	81 (24.6)	<5	<5	7 (14.0)
2	23 (43.4)	37 (13.4)	60 (18.2)	15 (57.7)	14 (58.3)	29 (58.0)
3 or more	8 (15.1)	7 (2.5)	15 (4.6)	8 (30.8)	6 (25.0)	14 (28.0)
Beginning of self-reported depression^a^						
Before pregnancy	20 (37.7)	39 (14.1)*	59 (17.9)	21 (80.8)	20 (83.3)	41 (82.0)
Pregnancy	4 (7.5)	−	4 (1.2)	<5	−	<5
Postpartum	15 (28.3)	28 (10.1)	43 (13.1)	<5	<5	<5
Beginning of self-reported anxiety^b^						
Before pregnancy	13 (24.5)	25 (9.1)	38 (11.6)	17 (65.4)	17 (65.4)	35 (70.0)
Pregnancy	6 (11.3)	14 (5.1)	20 (6.1)	<5	<5	<5
Postpartum	20 (37.7)	35 (12.7)	55 (16.7)	<5	−	<5
EPDS score, mean (s.d.)	13.1 (5.3)	14.3 (3.7)*	14.1 (4.0)	14.9 (4.3)	13.4 (2.7)	14.2 (3.7)
Perceived stigma for mental illness, mean (s.d.)	3.2 (0.9)	3.0 (1.0)	3.1 (1.0)	1.98 (0.98)	2.0 (0.95)	1.99 (0.98)

EPDS, Edinburgh Perinatal Depression Scale.

a. Only for women self-reporting the presence of depression.

b. Only for women self-reporting the presence of anxiety disorder.

*P < 0.05, **P < 0.001 for the comparison of the distribution of maternal characteristics by treatment status groups in each sample.

Concerning the Portuguese sample, women receiving no treatment more often reported not having any mental illness despite presenting with current active symptoms of depression. For most women who self-reported having depression, they reported that the illness began before pregnancy. A significantly higher proportion of women on no treatment reported that the depression began before pregnancy, compared with women receiving treatment. In Norway, there were no substantial differences in the distribution of clinical characteristics by treatment status. Results were consistent in the pregnant and postpartum groups.

### Treatment option uptake in the Portuguese and Norwegian samples

Table 3 presents the treatment option uptake among perinatal women receiving treatment in the Portuguese and Norwegian samples, and the difference between samples. In the Portuguese sample, the majority of women were receiving psychological treatment (individual psychotherapy), either as a sole option (45.2%) or combined with pharmacological treatment (21.4%), and for most women treatment started before pregnancy. In the Norwegian sample, most women were receiving only pharmacological (36.5%) or combined treatment (35.4%), and the treatment started before pregnancy. Compared with the Portuguese sample, a higher proportion of Norwegian perinatal women started treatment (either psychological or pharmacological) before pregnancy (difference in proportion: 29.9%–32.7%).

**Table 3:** Treatment options among perinatal women receiving treatment in both Portuguese and Norwegian samples

	Portuguese sample *(n =* 84)	Norwegian sample (*n* = 96)	Absolute difference between samples, % [95% CI]
	*n* (%)	*n* (%)	
Treatment options			
Only psychological	38 (45.2)	27 (28.1)	17.1 [−6.8 to 37.6]
Only pharmacological	28 (33.3)	35 (36.5)	3.2 [−19.9 to 25.2]
Combined treatment	18 (21.4)	34 (35.4)	14.0 [−12.8 to 35.2]
Psychological treatment^a^			
Individual therapy	58 (100.0)	56 (91.9)	8.4 [0.5–18.6]*
Group therapy	−	5 (8.2)	Not applicable
Beginning of psychological treatment^a^			
Before pregnancy	23 (41.1)	45 (73.8)	32.7 [8.1–53.1]**
During pregnancy	20 (35.8)	11 (18.0)	17.8 [−16.3 to 43.0]
During postpartum	13 (23.3)	5 (8.2)	15.1 [−31.2 to 43.3]
Pharmacological treatment^b^,^c^			
Sertraline	24 (52.2)	21 (30.4)	21.8 [−6.7 to 45.6]
Escitalopram	11 (23.9)	34 (49.3)	25.4 [−8.1 to 48.0]
Fluoxetine	6 (13.0)	13 (18.8)	5.8 [−36.2 to 35.1]
Venlafaxine	5 (10.9)	9 (13.0)	2.1 [−42.7 to 36.0]
Other	3 (6.5)	14 (20.3)	13.8 [−42.8 to 40.7]
Beginning of pharmacological treatment^b^			
Before pregnancy	25 (55.6)	59 (85.5)	29.9 [9.2–50.0]**
During pregnancy	18 (40.0)	7 (10.1)	29.9 [−11.6 to 53.9]
During postpartum	<5	<5	Not applicable

a. Only for women who currently have psychological treatment (sole or combined).

b. Only for women who currently have pharmacological treatment (sole or combined).

c. Some women reported more than one option.

*P < 0.05, **P < 0.001 for the comparison of the distribution of maternal characteristics between the Norwegian and Portuguese samples.

### Maternal factors associated with treatment uptake in Portugal and Norway

Table 4 presents the univariate and multivariate logistic regression models examining the maternal factors (sociodemographic, health and clinical-related factors) associated with perinatal treatment uptake, both in Portugal and Norway.

**Table 4:** Univariate and multivariate logistic regressions for sociodemographic, health and clinical factors associated with women's treatment options

Total perinatal women	Portuguese sample				Norwegian sample			
	Dependent variable: treatment status (0 = no treatment, 1 = on any treatment)							
	Univariate analyses		Multivariate analyses		Univariate analyses		Multivariate analyses	
	Odds ratio (95% CI)	*P*-value	Odds ratio (95% CI)	*P*-value	Odds ratio (95% CI)	*P*-value	Odds ratio (95% CI)	*P*-value
Age	0.98 [0.93–1.03]	0.406	0.98 [0.92–1.04]	0.517	1.03 [0.96–1.09]	0.440	1.01 [0.94–1.09]	0.749
Marital status	0.99 [0.39–2.50]	0.978			1.95 [0.37–10.35]	0.433		
Educational level	0.71 [0.39–1.28]	0.254	0.54 [0.25–1.16]	0.112	0.83 [0.43–1.59]	0.577	0.81 [0.38–1.70]	0.570
Occupational status	1.70 [0.36–8.09]	0.505			1.32 [0.66–2.63]	0.426		
Number of children	0.38 [0.20–0.73]	0.004	0.99 [0.35–2.84]	0.985	1.77 [0.92–3.39]	0.085	1.66 [0.83–3.31]	0.148
Perinatal status	2.88 [1.70–4.89]	<0.001	1.96 [0.86–4.44]	0.109	1.32 [0.69–2.56]	0.414		
Pregnancy planning	1.54 [0.93–2.53]	0.093	1.46 [0.78–2.71]	0.235	1.21 [0.63–2.32]	0.561		
Depressive symptoms (EPDS)	0.91 [0.85–0.97]	0.002	0.86 [0.80–0.92]	<0.001	1.02 [0.94–1.12]	0.540		
Self-reported psychopathology	3.69 [2.71–5.03]	<0.001	4.35 [3.05–6.19]	<0.001	1.40 [0.90–2.16]	0.133	1.41 [0.90–2.20]	0.138
Perceived stigma	1.13 [0.88–1.45]	0.341			0.82 [0.61–1.09]	0.176	0.85 [0.63–1.15]	0.294

Marital status: 0 = married/living together, 1 = single/divorced); educational level: 0 = university, 1 = high school or less; occupational status: 0 = occupied, 1 = unemployed, student or houseworker; number of children: 0 = no prior children, 1 = having prior children; perinatal status: 0 = postpartum, 1 = pregnancy; pregnancy planning: 0 = yes, 1 = no; self-reported psychopathology: number of mental diseases self-reported, from 0 to 3 or more. EPDS, Edinburgh Perinatal Depression Scale.

In the multivariate models with the Portuguese sample, having higher depressive symptoms was associated with a decreased likelihood of being on treatment, whereas self-reported psychopathology was found to be significantly associated with higher likelihood of being on treatment. In the Norwegian sample of perinatal women, we found no maternal factors to be significantly associated with treatment uptake in the multivariate model.

## Discussion

The present exploratory study contributes with new knowledge about treatment option uptake and related factors in perinatal women with active symptoms of depression, both in Portugal and Norway. By targeting this underexamined topic, our results allow us to highlight clinically relevant knowledge about the extent of treatment uptake in different countries, as well as to identify the women at higher risk of not receiving treatment in the presence of active symptoms of depression in the perinatal period.

First, in both samples there is a high proportion of perinatal women that are not receiving any treatment despite presenting active symptoms of depression (>50%), although the proportion is higher in Portugal (79.8% *v*. 53.9% in Norway). The more elevated proportion in Portugal than in Norway could be explained by at least two factors: different recruitment strategies used in the two countries and availability of clinical practice guidelines for perinatal depression management in Norway, but not in Portugal.^17^ The lack of systematic screening procedures in both countries may also play a role. At the same time, the substantial lack of treatment uptake observed in this study may, at least in part, reflect women's barriers to help-seeking during the perinatal period.^20^ Research has consistently identified important barriers to perinatal women's help-seeking, such as inadequate knowledge, poor mental health literacy, difficulty in distinguishing the normal reactions of transition to parenthood from psychopathological symptoms and knowing where to seek help.^29,30^ Although the low proportion of perinatal women receiving treatment in Portugal is congruent with prior studies,^15^ identifying barriers to help-seeking that could explain our findings was not within the scope of this study.

Second, we found no differences in sociodemographic, health and clinical variables between women receiving any treatment and women not receiving treatment in Norway, and these results were consistent when pregnant women and recent mothers were considered separately. Instead, Portuguese women not receiving any treatment were more frequently in the postpartum period and had prior children. It is possible that postpartum women may feel more stigma in relation to treatment uptake for mental health problems^9^ when compared with pregnant women, as they may fear being labelled as ‘bad’ or ‘incompetent’ mothers, or that their babies may be taken away.^30,31^ In addition, it is possible that women with prior children may be faced with more practical barriers (lack of time, childcare needs, financial barriers) that prevent them from seeking professional help.^29^ It is important to note that although in Norway most women with active symptoms of depression identify themselves as having a mental illness, in Portugal, more than half of women not receiving treatment did not report having mental health problems. One hypothesis is that poor mental health literacy levels among perinatal Portuguese women^32^ could have contributed to these results, as it may hinder their ability to identify the presence of depressive symptoms.^15,31,33^ Differences in eligibility criteria for the study population in Norway versus Portugal may largely explain this result. In Norway, women who had been offered antidepressant treatment in the past 5 years constituted the target population, whereas no such eligibility criterion was applied in Portugal.

Third, when considering treatment options, receiving psychological treatment was found to be the most frequent option in the Portuguese sample, whereas receiving pharmacological treatment was the most frequent option in the Norwegian sample. One possible explanation for this difference may be the recruitment strategy in both countries (self-selected sample in Portugal versus women being offered antidepressant treatment in Norway in the past 5 years). Hence, our results from the Portuguese samples may not be completely representative of the pattern of treatment in Portugal.

One key finding is the significantly higher proportion of women in the Norwegian sample who had started both pharmacological and psychological treatment before pregnancy, when compared with the Portuguese sample. In the latter sample, treatment was more often started in pregnancy or postpartum. One possible explanation for these results is that in Portugal, the low rates of help-seeking for mental health problems also occur before pregnancy (in the general population), because the Portuguese mental health system has an insufficient provision of community-based mental health services.^34^ As previously discussed, the Norwegian results are most likely attributable to the applied eligibility criteria possibly targeting moderate-to-severe mental illness cases.

Finally, when considering the predictors of receiving any treatment for perinatal depression, different results were found in both samples: in Norway, no significant sociodemographic or clinical predictors emerged; in Portugal, women currently reporting lower depressive symptoms and self-reporting more mental illnesses had a higher likelihood of being on any treatment. On the one hand, these results suggest that other variables – psychological and interpersonal variables, rather than only sociodemographic and clinical variables – may be involved in the decision-making process of treatment uptake in both countries, and that further studies should explore these hypotheses. On the other hand, in the Portuguese sample, it seems that when women self-identify as having more psychopathological illnesses, they are more prone to uptake treatment, suggesting the important role of recognition of symptoms and mental health literacy.^32^ This latter association was generally present also in the Norwegian sample, but the small sample size produced a broad 95% confidence interval with a borderline *P*-value.

### Limitations

The study has some limitations that need to be acknowledged when interpreting the results. First, the recruitment methods in both countries followed different strategies (self-selected sample in Portugal versus women who had been offered antidepressant treatment in the past 5 years in Norway). This may have influenced the comparisons between countries and may hinder the representativeness of the general childbearing population of women with mental illnesses in both countries. One prior study, using the same Norwegian data, applied survey weight adjustment (based on the most recent data from the Norwegian Health Directorate regarding the proportion of female patients having had contact with psychiatric clinics in all health regions), which affected results only minimally.^35^ The two questionnaires in Norway and Portugal were generally identical, allowing uniform data collection and direct comparability of results. In addition, in both samples, the questionnaires were administered through a web-based platform, which may have prevented women with low digital literacy from participating in the study. However, the validity of web-based recruitment methods is now well acknowledged,^36^ and both countries have a very high internet penetration rate among women of childbearing age. Use of an electronic questionnaire and multiple recruitment strategies did not permit calculation of a conventional response rate, and bias owing self-selection cannot be ruled out. However, among the women who expressed their willingness to participate in the study in Norway and Portugal, the response rate was satisfactory (66% and 67%, respectively). Data collection was conducted under different waves of the COVID-19 pandemic, which may have produced an underestimation of the true rate of treatment uptake, given the lower possibility of women's access to healthcare. In Norway, there were no lockdown measures applied at the time of data collection.^37^ In Portugal, the time of data collection coincided with the beginning of vaccination and the gradual withdrawal of the restriction measures (e.g. the presence of companion during prenatal appointments and labor^38^). In addition, the study was intended to be an exploratory study providing preliminary insights about perinatal treatment uptake in both countries, and that may set the stage for future studies.

Second, the presence of active depressive symptoms and mental illness was self-reported by women in both samples (and not assessed by a health professional), which may have prevented some women from being included in the study, given the lack of symptom recognition.^32^ The self-reported nature of the questions about mental illnesses may have led to their underestimation, since underreporting is most often seen among individuals who have less severe illness or who have not received treatment.^39^ However, the EPDS is a validated, perinatal-specific screening tool.^23,24^ Third, the sample size for women in some categories (sociodemographic and clinical characteristics) was low, compromising the statistical analyses and the ability to identify factors associated with the specific treatment option uptake. The cross-sectional nature of the study does not allow for examination of the variability of treatment uptake over time during the perinatal period. Finally, the study did not address the diversity of stigmatising attitudes, and focused only on women's perceived stigma related to mental illness.

Despite these limitations, the present study provides important insights on treatment uptake in Portugal and Norway, two European countries with different practice guidelines for perinatal depression management. Our results can contribute to a better understanding of which groups of perinatal women are at higher risk of not receiving treatment for their depressive symptoms, and therefore highlight the need to develop and implement strategies to improve help-seeking behaviours among this population.

## Data Availability

The data that support the findings of this study for Portugal are available from the corresponding author, M.B., upon reasonable request. The data that support the findings of this study for Norway can only be made available after approval by the Ethics Committee and the Data Protection Agency in Norway.

## Supplementary material

For supplementary material accompanying this paper visit http://doi.org/10.1192/bjo.2023.56. click here to view supplementary material
