# Supplementary material for: Treatment options and their uptake among women with symptoms of perinatal depression: exploratory study in Norway and Portugal
Source: BJPsych Open. 2023 May 4;9(3):e77. doi: 10.1192/bjo.2023.56 (PMC10228243; doi:10.1192/bjo.2023.56)
Supplement: Supplementary file 1 [file bjosup.zip › S205647242300056Xsup001.docx]

**Supplement 1:** Recruitment strategies used in the study, including psychiatric clinics, websites, apps and social media

| **Recruitment strategy 1:**  Informative brochures about the study available at the site, which could be taken freely by the participants | |
| --- | --- |
| *Site specification/type* | *Site location(s)* |
| Psychiatric outpatient polyclinics | Østre Agder, Lister, Flekkefjord, Solvang, Strømme (Agder region); Lørenskog (Viken region); North of Norway (Tromsø and surrounding areas) |
| Outpatient polyclinic for anxiety | Flekkefjord (Agder region)) |
| Specialized outpatient polyclinic of psychosomatics and trauma | Lundsiden (Agder region)) |
| Psychiatric hospital ward | Hospital of South Norway (Sørlandet sykehus), Akershus Universitetssykehus HF in Lørenskog (Viken region) |
| Regional Section for Eating Disorders | Oslo University Hospital, Villa Sult in Oslo |
| Public prenatal and postnatal care health clinics | Oslo (Grunerløkka district, Østensjø), Stavanger, Bergen, Trondheim, Tromsø, Ås, Tingvoll, Hareid |
| **Recruitment strategy 2:**  Information about the study on selected pregnancy-motherhood specific websites, as well as medically oriented websites in Norwegian language, social media and pregnancy forums | |
| General pregnancy / motherhood specific websites or Facebook page | www.ammehjelp.no (breastfeeding support network), www.altformamma.no (general website for mothers), |
| Medical-specific websites | www.hjelptilhjelp.no (portal for mental health); www.nhi.no (health portal for healthcare personnel and lay persons); www.tryggmammamedisin.no (National medicines information centre for pregnant and breastfeeding women) |
| Social media | Facebook (featured ads and posts in pregnancy-related and mental health-related pages and groups), Twitter, featured google ads |
| Pregnancy forums | Kvinneguide (forum for women in general) |
| **Recruitment strategy 3:**  Information about the study distributed by patient organizations and peers via social media | |
| Social media | Organization «Psykisk helse» (Mental health) via Twitter; organization “Landsforening1001dager» (perinatal mental health organization) via their Facebook page; “Norske Kvinners Sanitetsforening” (Women association of Norway) via their Facebook and twitter page |
| **Recruitment strategy 4:**  Information about the study distributed to users of pregnancy-specific or women-specific apps | |
| Apps | “Clue”, an app to track ovulation and pregnancy planning; “Helseoversikt”, an app recommended by all prenatal and postnatal health centres in Norway to track health appointments for mother and child, and other health-related information on pregnancy, motherhood and infant care |
