# Supplementary material for: Treatment options and their uptake among women with symptoms of perinatal depression: exploratory study in Norway and Portugal
Source: BJPsych Open. 2023 May 4;9(3):e77. doi: 10.1192/bjo.2023.56 (PMC10228243; doi:10.1192/bjo.2023.56)
Supplement: Supplementary file 1 [file bjosup.zip › S205647242300056Xsup002.docx]

**Healthx2 - Patient-centered approaches to aid women’s decision-making and set priorities in perinatal antidepressant research**

electronic questionnaire

INFORMATION ABOUT YOURSELF

1. **In which region/province do you live?**

Region:

1. **Please specify your current pregnancy status**.

🞎 I am pregnant

🞎 I have recently given birth (in the last year)

🞎 I have given birth in the last 5 years

1. (If yes “I am pregnant): **In which pregnancy week are you?**

From 1 to 44

1. (If yes to “I have recently given birth (in the last year)” or to “I have given birth in the last 5 years”): **How old is your child?**

🞎 less than 1 month

🞎 1-3 months

🞎 4-6 months

🞎 7-9 months

🞎 10-12 months

🞎 1-5 years

1. (If yes to “I have recently given birth (in the last year)” or to “I have given birth in the last 5 years”): **Are you currently breastfeeding or have you breastfed your child?**

🞎 Yes 🞎 No

1. (If yes “Are you currently breastfeeding or have you breastfed your child?): **What kind of breastfeeding?**

🞎 Exclusive with breastmilk from 0 to 4-6 months 🞎 Partial with formula/milk from 0 to 4-6 months

1. **Have you been pregnant before? (This also applies to pregnancy that ended in abortion, miscarriage or fetal death)**

🞎 Yes 🞎 No

1. **How many children do you have now?**

🞎 None 🞎 1 🞎 2 🞎 more than 2

1. **What is your marital status?**

🞎 Married 🞎 Cohabitant 🞎 Single 🞎 Divorced/Separated

🞎 Other/Please specify:______________________________

1. **What is the highest education you have completed?**

🞎 Primary school (10 years of education)

🞎 High-school (11-13 years of education)

🞎 University / college

🞎 Other/Please specify: _________________________________

1. **Your age (in years)?**

From 15 to 55:

1. **Is Norwegian your mother tongue?** 🞎 Yes 🞎 No
2. **How tall are you (in cm)**?____________________
3. (If yes to “I am pregnant” or “I have recently given birth (in the last year)” or “I have given birth in the last 5 years”): **What was your weight at the time of conception (in kg)? ___________________________**
4. (If yes to “I have recently given birth (in the last year)” or to “I have given birth in the last 5 years” or “I am planning a pregnancy”): **What is your current weight now (in kg)? _____________________________**

INFORMATION ABOUT YOUR FUTURE PREGNANCY

(If yes to “I am planning a pregnancy”), for questions 16-19:

1. **When are you planning to get pregnant?**

🞎 Within the next 6 months 🞎 Within one year 🞎 More than 1 year from now

1. **Are you currently trying to conceive?**

🞎 Yes 🞎 No 🞎 Other, please specify___________________

1. **Do you smoke cigarettes?**

🞎 No 🞎 Sometimes 🞎 Daily

1. **What is your current work situation?**

🞎 Student

🞎 Homemaker

🞎 Health care personnel, i.e., physician, nurse, or pharmacist

🞎 Employed in another sector

🞎 Job seeker

🞎 None of the above, specify:_________________________________________________

INFORMATION ABOUT YOUR CURRENT OR LATEST PREGNANCY

(If yes to “I am pregnant” or “I have recently given birth (in the last year)” or “I have given birth in the last 5 years”) for questions 20-23:

1. **Was your pregnancy planned?**

🞎 Yes 🞎 No, but it was not completely unexpected 🞎 No, it was not planned

1. **Did you drink any alcohol after finding out that you were pregnant?**

🞎 Yes 🞎 No 🞎 cannot remember

1. **Did you smoke after finding out you were pregnant?**

🞎 No 🞎 Sometimes 🞎 Daily

1. **What was your work situation when you became pregnant?**

🞎 Student

🞎 Homemaker

🞎 Health care personnel, i.e., physician, nurse, or pharmacist

🞎 Employed in another sector

🞎 Job seeker

🞎 None of the above, specify:_________________________________________________

HOW YOU ARE DOING AND YOUR MEDICATION USE

The next questions are about your well-being and your use of medication.

1. (If yes to “I am planning a pregnancy”). **Do you have you or have you had any of the following mental illnesses or health problems? If yes, check the box when you have experienced the illnesses.**

|  | More than 1 year ago | Within the last year | Currently |
| --- | --- | --- | --- |
| 🞎 Depression | 󠄿 | 󠄿 | 󠄿 |
| 🞎 Anxiety | 󠄿 | 󠄿 | 󠄿 |
| 🞎 Obsessive Compulsive disorder | 󠄿 | 󠄿 | 󠄿 |
| 🞎 Eating disorder (for example bulimia, anorexia, binge eating) | 󠄿 | 󠄿 | 󠄿 |
| 🞎 Other mental illness | 󠄿 | 󠄿 | 󠄿 |
| 🞎 No mental illness | 󠄿 | 󠄿 | 󠄿 |

1. (If yes to “I am pregnant” or “I have recently given birth (in the last year)” or “I have given birth in the last 5 years”). **Have you or have you had any of the following mental illnesses or health problems in the period around your pregnancy? If yes, check the box when you have experienced the illnesses. Please choose the alternatives that apply to you**.

|  | More than 1 year before pregnancy | 1 year or less before pregnancy | In 1^st^ trimester | In 2^nd^ trimester | In 3^rd^ trimester | 0 to 6 months after birth | 7 to 12 months after birth |
| --- | --- | --- | --- | --- | --- | --- | --- |
| 🞎 Depression | 󠄿 | 󠄿 | 󠄿 | 󠄿 | 󠄿 | 󠄿 | 󠄿 |
| 🞎 Anxiety | 󠄿 | 󠄿 | 󠄿 | 󠄿 | 󠄿 | 󠄿 | 󠄿 |
| 🞎 Obsessive Compulsive disorder | 󠄿 | 󠄿 | 󠄿 | 󠄿 | 󠄿 | 󠄿 | 󠄿 |
| 🞎 Eating disorder (for example bulimia, anorexia, binge eating) | 󠄿 | 󠄿 | 󠄿 | 󠄿 | 󠄿 | 󠄿 | 󠄿 |
| 🞎 Other mental illness | 󠄿 | 󠄿 | 󠄿 | 󠄿 | 󠄿 | 󠄿 | 󠄿 |
| 🞎 No mental illness | 󠄿 | 󠄿 | 󠄿 | 󠄿 | 󠄿 | 󠄿 | 󠄿 |

1. (If yes to “I am planning a pregnancy”). **If you have recently received or receive now psychological treatment (e.g. therapy) for your mental illness, please specify which and when**:

🞎 Yes, I receive or have recently received psychological treatment

🞎 No, I do not receive or have not recently received any psychological treatment

1. (If yes in question 26). **If yes, what kind? (for example individual psychotherapy, group therapy, counselling session)___________________________________________________________________**
2. (If yes in question 26): **If yes, when?**

| More than 1 year ago | Within the last year | Currently |
| --- | --- | --- |
| 󠄿 | 󠄿 | 󠄿 |

1. (If yes to “I am pregnant” or “I have recently given birth (in the last year)” or “I have given birth in the last 5 years”). **If you have recently received or receive now psychological treatment (e.g. therapy) for your mental illness, please specify which and when**:

🞎 Yes, I receive or have recently received psychological treatment

🞎 No, I do not receive or have not recently received any psychological treatment

1. (If “Yes in question 29) **If yes, what kind? (for example individual psychotherapy, group therapy, counselling session)___________________________________________________________________**
2. (If “Yes in question 29) **If yes, when?**

| More than 1 year before pregnancy | 1 year or less before pregnancy | In 1^st^ trimester | In 2^nd^ trimester | In 3^rd^ trimester | 0 to 6 months after birth | 7 to 12 months after birth |
| --- | --- | --- | --- | --- | --- | --- |
| 󠄿 | 󠄿 | 󠄿 | 󠄿 | 󠄿 |  |  |

1. **During the past month: have you often been bothered by feelings of sadness, depression or hopelessness?**

🞎 Yes 🞎 No

1. **During the past month: have you often been bothered by having less interest in things or less pleasure in doing things?**

🞎 Yes 🞎 No

The next 10 questions are about how you have been doing it for the last 7 days. There are no right or wrong answers. We are only interested in your personal views. (Tick only one box per question)

*In this section of the questionnaire - questions 34-43 -* ***the Edinburgh Postnatal Depression Scale (EPDS)*** *was presented (Cox J, Holden J, Sagovsky R. Detection of postnatal depression. Development of the 10-item edinburgh postnatal depression scale. The British Journal of Psychiatry. 1987 June 1, 1987;150(6):782-6).*

The next questions are about your weight and weight control.

(If yes to “I am planning a pregnancy”), for questions 44-45

1. **Do you think you are overweight now that you plan a pregnancy?**

🞎 Yes, a lot

🞎 Yes, little

🞎 No

1. **Are you or have you been worried about putting on more weight than necessary during a pregnancy?**

🞎 Yes, very worried

🞎 Somewhat worried

🞎 No, not especially worried

1. (If yes “I am pregnant): **Do you think you were overweight just before this pregnancy?**

🞎 Yes, a lot

🞎 Yes, little

🞎 No

1. (If yes “I am pregnant): **Are you or have you been worried about putting on more weight than necessary during this pregnancy?**

🞎 Yes, very worried

🞎 Somewhat worried

🞎 No, not especially worried

1. (If yes to “I have recently given birth (in the last year)” or to “I have given birth in the last 5 years”): **Do you think you were overweight just in this period:**

|  | Yes, a lot | Yes, a little | No |
| --- | --- | --- | --- |
| Just before the pregnancy | 🞎 | 🞎 | 🞎 |
| During pregnancy | 🞎 | 🞎 | 🞎 |
| The first 12 months after birth | 🞎 | 🞎 | 🞎 |

1. (If yes to “I have recently given birth (in the last year)” or to “I have given birth in the last 5 years”): **Are you or have you been worried about putting on more weight than necessary in this period:**

|  | Yes, very worried | Somewhat worried | No, not especially worried |
| --- | --- | --- | --- |
| During the last pregnancy | 🞎 | 🞎 | 🞎 |
| The first 12 months after my latest birth | 🞎 | 🞎 | 🞎 |

1. **Has anyone said that you were too thin while you felt that you were overweight during the last 2 years?**

🞎 Yes, often

🞎 Yes, occasionally

🞎 No

(If yes to “I am planning a pregnancy”), for questions 51-52

1. **Have you ever felt that you lost control while eating and were not able to stop before you have eaten far too much?**

|  | Last 6 months | Currently |
| --- | --- | --- |
| No | 🞎 | 🞎 |
| Infrequently | 🞎 | 🞎 |
| Yes, at least once a week | 🞎 | 🞎 |

1. **Have you used any of the following methods to control your weight during the last 6 months?**

| Vomiting | 🞎  Atleast once a week | 🞎  Seldom | 🞎  Never |
| --- | --- | --- | --- |
| Laxatives | 🞎  Atleast once a week | 🞎  Seldom | 🞎  Never |
| Fasting | 🞎  Atleast once a week | 🞎  Seldom | 🞎  Never |
| Hard physical exercise | 🞎  Atleast once a week | 🞎  Seldom | 🞎  Never |

(If yes to “I am pregnant” or “I have recently given birth (in the last year)” or “I have given birth in the last 5 years”), for questions 53-55

1. **Have you ever felt that you lost control while eating and were not able to stop before you have eaten far too much? (remember to choose only the period relevant for you)**

|  | Last 6 months before this pregnancy | During pregnancy | The first 12 months after birth |
| --- | --- | --- | --- |
| No | 🞎 | 🞎 | 🞎 |
| Infrequently | 🞎 | 🞎 | 🞎 |
| Yes, at least once a week | 🞎 | 🞎 | 🞎 |

1. **Have you used any of the following methods to control your weight during the last 6 months before pregnancy?**

| Vomiting | 🞎  Atleast once a week | 🞎  Seldom | 🞎  Never |
| --- | --- | --- | --- |
| Laxatives | 🞎  Atleast once a week | 🞎  Seldom | 🞎  Never |
| Fasting | 🞎  Atleast once a week | 🞎  Seldom | 🞎  Never |
| Hard physical exercise | 🞎  Atleast once a week | 🞎  Seldom | 🞎  Never |

1. **Have you used any of the following methods to control your weight during pregnancy?**

| Vomiting | 🞎  Atleast once a week | 🞎  Seldom | 🞎  Never |
| --- | --- | --- | --- |
| Laxatives | 🞎  Atleast once a week | 🞎  Seldom | 🞎  Never |
| Fasting | 🞎  Atleast once a week | 🞎  Seldom | 🞎  Never |
| Hard physical exercise | 🞎  Atleast once a week | 🞎  Seldom | 🞎  Never |

(If yes to “I have recently given birth (in the last year)” or to “I have given birth in the last 5 years”):

1. **Have you used any of the following methods to control your weight during the first 12 months after pregnancy?**

| Vomiting | 🞎  Atleast once a week | 🞎  Seldom | 🞎  Never |
| --- | --- | --- | --- |
| Laxatives | 🞎  Atleast once a week | 🞎  Seldom | 🞎  Never |
| Fasting | 🞎  Atleast once a week | 🞎  Seldom | 🞎  Never |
| Hard physical exercise | 🞎  Atleast once a week | 🞎  Seldom | 🞎  Never |

1. **Is it important for your self-image that you maintain a certain weight?**

🞎 Yes, very important

🞎 Yes, quite important

🞎 No, not especially important

The next questions are about your views about use of antidepressant medication during pregnancy.

1. **Have you previously taken or are you currently taking antidepressant medications?**

🞎 Yes, I have previously taken and/or I am currently taking antidepressant medication

🞎 No

1. **What is your preference regarding treatment with antidepressant during pregnancy?**

🞎 Continue treatment with the same antidepressant(s)

🞎 Switch to another antidepressant

🞎 Discontinue use of the antidepressant

🞎 Reduce the dose of the antidepressant

🞎 No preference

🞎 Other, specify:_________________________________

1. **Do you think that antidepressants can be safely used in all phases of pregnancy? (You can choose multiple answers)**

🞎 No

🞎 A woman should receive tailored counselling to facilitate her decision-making whether to take medications or not

🞎 Use has to be stopped because it is harmful to the unborn child

🞎 Use must not be discontinued, because this can be harmful for maternal mental health

🞎 No preference

🞎 Other, specify:_________________________________

1. (If yes to “I am planning a pregnancy”). **The next questions are about your treatment with antidepressant medication. If you are taking now or have taken antidepressant medication for your mental illness in the last 6 months, please select the relevant antidepressants from the list below and when you used them. If you did not take antidepressant, you can skip this question**.

|  | Last 6 months | Now |
| --- | --- | --- |
| 🞎 Fluoxetine (incl. Fontex, etc) | 󠄿 | 󠄿 |
| 🞎 Fluoxetine (incl. Fluoxetin Mylan, Fluoxetine Orion, Fontex) | 󠄿 | 󠄿 |
| 🞎 Citalopram (incl. Cipramil Farmagon, Cipramil Lundbeck, Citalopram Sandoz) | 󠄿 | 󠄿 |
| 🞎 Escitalopram (incl. Cipralex Farmagon, Cipralex Lundbeck, Escitalopram Actavis) | 󠄿 | 󠄿 |
| 🞎 Paroxetine (incl. Seroxat, Paroxetin Actavis, Paroxetin Farmagon) | 󠄿 | 󠄿 |
| 🞎 Sertraline (incl. Sertralin HEXAL, Zoloft, Sertraline Accord) | 󠄿 | 󠄿 |
| 🞎 Fluvoxamine (incl. Fevarin Mylan, Fevarin Orifarm) | 󠄿 | 󠄿 |
| 🞎 Venlafaxine (incl. Efexor, Venorion, Venlazid, Venlafaxin Bluefish) | 󠄿 | 󠄿 |
| 🞎 Duloxetine (incl. Cymbalta, Duloxetin Pensa, Duloxetine Mylan) | 󠄿 | 󠄿 |
| 🞎 Mirtazapine (incl. Remeron, Mirtazapin Bluefish) | 󠄿 | 󠄿 |
| 🞎 Reboxetine (incl. Edronax) | 󠄿 | 󠄿 |
| 🞎 Mianserin (incl. Mianserin Mylan, Tolvon) | 󠄿 | 󠄿 |
| 🞎 Amitriptyline (incl. Anafranil, Klomipramin Mylan) | 󠄿 | 󠄿 |
| 🞎 Clomipramine (incl. Anafranil, Klomipramin Mylan) | 󠄿 | 󠄿 |
| 🞎 Trimipramine (incl. Surmontil) | 󠄿 | 󠄿 |
| 🞎 Nortriptyline (incl. Noritren) | 󠄿 | 󠄿 |
| 🞎 Doxepine (inkl. Sinequan) |  |  |

1. (If yes to “I am pregnant” or “I have recently given birth (in the last year)” or “I have given birth in the last 5 years”). **The next questions are about your treatment with antidepressant medication. If you are taking now or have taken antidepressant medication for your mental illness in the period around pregnancy, please select the relevant antidepressants from the list below and when you used them. (Remember to choose relevant alternatives). If you did not take antidepressant, you can skip this question**.

|  | More than 6 months before pregnancy | 6 months or less before pregnancy | 1^st^ trimester | 2^nd^ trimester | 3^rd^ trimester | 0 to 6 months after birth | 7 to 12 months after birth |
| --- | --- | --- | --- | --- | --- | --- | --- |
| 🞎 Fluoxetine (incl. Fluoxetin Mylan, Fluoxetine Orion, Fontex) | 󠄿 | 󠄿 | 󠄿 | 󠄿 | 󠄿 | 󠄿 | 󠄿 |
| 🞎 Citalopram (incl. Cipramil Farmagon, Cipramil Lundbeck, Citalopram Sandoz) | 󠄿 | 󠄿 | 󠄿 | 󠄿 | 󠄿 | 󠄿 | 󠄿 |
| 🞎 Escitalopram (incl. Cipralex Farmagon, Cipralex Lundbeck, Escitalopram Actavis) | 󠄿 | 󠄿 | 󠄿 | 󠄿 | 󠄿 | 󠄿 | 󠄿 |
| 🞎 Paroxetine (incl. Seroxat, Paroxetin Actavis, Paroxetin Farmagon) | 󠄿 | 󠄿 | 󠄿 | 󠄿 | 󠄿 | 󠄿 | 󠄿 |
| 🞎 Sertraline (incl. Sertralin HEXAL, Zoloft, Sertraline Accord) | 󠄿 | 󠄿 | 󠄿 | 󠄿 | 󠄿 | 󠄿 | 󠄿 |
| 🞎 Fluvoxamine (incl. Fevarin Mylan, Fevarin Orifarm) | 󠄿 | 󠄿 | 󠄿 | 󠄿 | 󠄿 | 󠄿 | 󠄿 |
| 🞎 Venlafaxine (incl. Efexor, Venorion, Venlazid, Venlafaxin Bluefish) | 󠄿 | 󠄿 | 󠄿 | 󠄿 | 󠄿 | 󠄿 | 󠄿 |
| 🞎 Duloxetine (incl. Cymbalta, Duloxetin Pensa, Duloxetine Mylan) | 󠄿 | 󠄿 | 󠄿 | 󠄿 | 󠄿 | 󠄿 | 󠄿 |
| 🞎 Mirtazapine (incl. Remeron, Mirtazapin Bluefish) | 󠄿 | 󠄿 | 󠄿 | 󠄿 | 󠄿 | 󠄿 | 󠄿 |
| 🞎 Reboxetine (incl. Edronax) | 󠄿 | 󠄿 | 󠄿 | 󠄿 | 󠄿 | 󠄿 | 󠄿 |
| 🞎 Mianserin (incl. Mianserin Mylan, Tolvon) | 󠄿 | 󠄿 | 󠄿 | 󠄿 | 󠄿 | 󠄿 | 󠄿 |
| 🞎 Amitriptyline (incl. Anafranil, Klomipramin Mylan) | 󠄿 | 󠄿 | 󠄿 | 󠄿 | 󠄿 | 󠄿 | 󠄿 |
| 🞎 Clomipramine (incl. Anafranil, Klomipramin Mylan) | 󠄿 | 󠄿 | 󠄿 | 󠄿 | 󠄿 | 󠄿 | 󠄿 |
| 🞎 Trimipramine (incl. Surmontil) | 󠄿 | 󠄿 | 󠄿 | 󠄿 | 󠄿 | 󠄿 | 󠄿 |
| 🞎 Nortriptyline (incl. Noritren) | 󠄿 | 󠄿 | 󠄿 | 󠄿 | 󠄿 | 󠄿 | 󠄿 |
| 🞎 Doxepine  (inkl. Sinequan) | 󠄿 | 󠄿 | 󠄿 | 󠄿 | 󠄿 | 󠄿 | 󠄿 |

1. (If yes to “I have recently given birth (in the last year)” or to “I have given birth in the last 5 years”): **Did you take antidepressant medications for your mental illness while breastfeeding?**

🞎 No, never

🞎 Yes, but the child received pumped milk when I took the medication(s)

🞎 Yes, irrespective of the use of antidepressant(s)

🞎 Yes, but I adapted the timing for breastfeeding according to the intake of the antidepressant

🞎 Cannot remember

🞎 Other, specify:_________________________________

1. (If yes to “I am pregnant” or “I have recently given birth (in the last year)” or “I have given birth in the last 5 years”). **Have you purposely stopped taking your prescribed antidepressant(s) during pregnancy?**

🞎 Yes 🞎 No 🞎 Cannot remember

1. (If yes in question 64) **Which antidepressant(s) was it?** ________________________________________________________
2. If yes in question 64) **Who recommended you to avoid antidepressant in pregnancy?**

🞎 Physician

🞎 Midwife

🞎 Pharmacy personnel

🞎 Family/friends

🞎 Internet

🞎 Nobody, my own initiative

(If yes to “I am pregnant” or “I have recently given birth (in the last year)” or “I have given birth in the last 5 years”).

1. **Was the dose of your prescribed antidepressant changed during pregnancy?**

🞎 Yes, increased

🞎 Yes, reduced

🞎 I stopped taking the medication

🞎 No

1. (If yes to “I am planning a pregnancy”): **On a scale from 0 (not at all) to 10 (very effective), how effective do you think your therapy with antidepressants is in treating your illness in general?**

0 1 2 3 4 5 6 7 8 9 10

1. If yes to “I am planning a pregnancy”): **On a scale from 0 (not at all) to 10 (very effective), how effective do you think your therapy with antidepressants will be in treating your illness during a future pregnancy?**

0 1 2 3 4 5 6 7 8 9 10

(If yes to “I am pregnant” or “I have recently given birth (in the last year)” or “I have given birth in the last 5 years”).

1. **On a scale from 0 (not at all) to 10 (very effective), how effective do you think your therapy with antidepressants is in treating your illness in general, regardless of your current or latest pregnancy?**

0 1 2 3 4 5 6 7 8 9 10

(If yes to “I am pregnant” or “I have recently given birth (in the last year)” or “I have given birth in the last 5 years”).

1. **On a scale from 0 (not at all) to 10 (very effective),** **how effective do you think your therapy with antidepressants was/is in treating your illness during your latest or current pregnancy?**

0 1 2 3 4 5 6 7 8 9 10

(If yes to “I am pregnant” or “I have recently given birth (in the last year)” or “I have given birth in the last 5 years”).

1. **If you are taking or have been taking other medications than antidepressants for your mental illness during the period of pregnancy, please choose relevant medications from the list below, and when you were using them.**

|  | 6 months or less before pregnancy | 1^st^ trimester | 2^nd^ trimester | 3^rd^ trimester | After birth |
| --- | --- | --- | --- | --- | --- |
| Paracetamol (for example Panodil, Pinex) |  |  |  |  |  |
| Opioid analgesics (for example Paralgin forte, Tramadol) |  |  |  |  |  |
| Lithium (Lithionit) |  |  |  |  |  |
| Antipsychotics (for example Zyprexa, Seroquel) |  |  |  |  |  |
| Anxiolytics (for example Valium, Sobril, Atarax) |  |  |  |  |  |
| Sleeping medications (for example Imovane, Stilnoct, Zolpidem) |  |  |  |  |  |

YOUR DECISION-MAKING ABOUT ANTIDEPRESSANT TREATMENT

The next questions are about your decision-making difficulties related to use of antidepressants in the period around pregnancy. There are no right or wrong answers. We are only interested in your personal views.

1. If yes to “I am planning a pregnancy”): **Which treatment option would you prefer during a future pregnancy?**

🞎 Pharmacological treatment with antidepressants

🞎 Non-pharmacological treatment

🞎 Combined non-pharmacological with antidepressants & therapy

🞎 No treatment

🞎 Unsure

1. (If yes to “I am pregnant” or “I have recently given birth (in the last year)” or “I have given birth in the last 5 years”). **Which treatment option do you prefer in pregnancy?**

🞎 Pharmacological treatment with antidepressants

🞎 Non-pharmacological treatment

🞎 Combined non-pharmacological with antidepressants & therapy

🞎 No treatment

🞎 Unsure

(Applicable to both questions 73 and 74): *In this section of the questionnaire,* ***the Decisional Conflict Scale (DCS)*** *was presented (O'Connor AM. Validation of a decisional conflict scale. Med Decis Making. 1995;15(1):25-30).*

YOUR PERCEPTION OF RISK DURING PREGNANCY AND WHILE BREASTFEEDING

1. Below is a list with various medications, food and other substances. Please indicate how harmful you think they are during pregnancy and lactation on a scale from 0 to 10, where 0 corresponds to ‘not harmful’ and 10 to ‘very harmful’. With the word “harmful”, we mean in relation to child longer-term development (for example autism, motor or language development, ADHD).

If you have not heard before about such substance, tick ‘unknown substance’.

|  | Unknown substance | 0 | 1 | 2 | 3 | 4 | 5 | 6 | 7 | 8 | 9 | 10 |
| --- | --- | --- | --- | --- | --- | --- | --- | --- | --- | --- | --- | --- |
| ***How dangerous are these during pregnancy for your child development?*** | ◦ | ◦ | ◦ | ◦ | ◦ | ◦ | ◦ | ◦ | ◦ | ◦ | ◦ | ◦ |
| Antidepressants | ◦ | ◦ | ◦ | ◦ | ◦ | ◦ | ◦ | ◦ | ◦ | ◦ | ◦ | ◦ |
| Antipsychotics | ◦ | ◦ | ◦ | ◦ | ◦ | ◦ | ◦ | ◦ | ◦ | ◦ | ◦ | ◦ |
| Anxiolytic benzodiazepines and sleeping drugs | ◦ | ◦ | ◦ | ◦ | ◦ | ◦ | ◦ | ◦ | ◦ | ◦ | ◦ | ◦ |
| Antiepileptics (e.g., valproate) |  |  |  |  |  |  |  |  |  |  |  |  |
| Cranberry | ◦ | ◦ | ◦ | ◦ | ◦ | ◦ | ◦ | ◦ | ◦ | ◦ | ◦ | ◦ |
| Maternal psychiatric disorder | ◦ | ◦ | ◦ | ◦ | ◦ | ◦ | ◦ | ◦ | ◦ | ◦ | ◦ | ◦ |
| Alcohol (*e.g. wine, beer, spirits*) | ◦ | ◦ | ◦ | ◦ | ◦ | ◦ | ◦ | ◦ | ◦ | ◦ | ◦ | ◦ |
| ***How dangerous are these while breastfeeding for your child development?*** | ◦ | ◦ | ◦ | ◦ | ◦ | ◦ | ◦ | ◦ | ◦ | ◦ | ◦ | ◦ |
| Antidepressants | ◦ | ◦ | ◦ | ◦ | ◦ | ◦ | ◦ | ◦ | ◦ | ◦ | ◦ | ◦ |
| Antipsychotics | ◦ | ◦ | ◦ | ◦ | ◦ | ◦ | ◦ | ◦ | ◦ | ◦ | ◦ | ◦ |
| Anxiolytic benzodiazepines and sleeping drugs | ◦ | ◦ | ◦ | ◦ | ◦ | ◦ | ◦ | ◦ | ◦ | ◦ | ◦ | ◦ |
| Antiepileptics (e.g., valproate) |  |  |  |  |  |  |  |  |  |  |  |  |
| Cranberry | ◦ | ◦ | ◦ | ◦ | ◦ | ◦ | ◦ | ◦ | ◦ | ◦ | ◦ | ◦ |
| Maternal psychiatric disorder | ◦ | ◦ | ◦ | ◦ | ◦ | ◦ | ◦ | ◦ | ◦ | ◦ | ◦ | ◦ |
| Alcohol (*e.g. wine, beer, spirits*) | ◦ | ◦ | ◦ | ◦ | ◦ | ◦ | ◦ | ◦ | ◦ | ◦ | ◦ | ◦ |

YOUR RELATIONSHIP TO YOUR DOCTOR AND TO YOUR PARTNER DURING PREGNANCY AND YOUR ATTITUDES TO MENTAL DISORDERS

Finally, here are some questions about your relationship with your doctor and your partner, as well as your attitudes towards mental illness. There are no right or wrong answers. We are only interested in your personal views. (Tick only one cross for each line)

1. **Your relationship with your doctor and your partner:**

*In this section of the questionnaire, selected items of the* “**Antidepressant Compliance Questionnaire” (ACQ)** tool were presented (K. Demyttenaere, et al. Development of an antidepressant compliance questionnaire. Acta Psychiatrica Scandinavica, 2004: 110; 3. 201-207).

b) **Your attitudes towards mental disorders:**

*In this section of the questionnaire, selected items of the* “**Indifference to stigma”** subscale were presented (Mackenzie et al. *An Adaptation and Extension of the Attitudes Toward Seeking Professional Psychological Help.* Journal of Applied Social Psychology. *2006:* 34; 11. 2410-2433).

**Thank you for your help!**
