# Supplementary material for: Treatment options and their uptake among women with symptoms of perinatal depression: exploratory study in Norway and Portugal
Source: BJPsych Open. 2023 May 4;9(3):e77. doi: 10.1192/bjo.2023.56 (PMC10228243; doi:10.1192/bjo.2023.56)
Supplement: Supplementary file 1 [file bjosup.zip › S205647242300056Xsup003.docx]

**women choose health – WOMEN’S DECISION-MAKING ON MENTAL HEALTH TREATMENTS IN THE PERINATAL PERIOD**

Online questionnaire

INFORMATION ABOUT YOURSELF

1. **In which region/province do you live?**

Region:

1. **How do you characterize the area where you live?**

🞎 Urban

🞎 Rural

1. **What is your marital status?**

🞎 Married/cohabitant

🞎 Single

🞎 Divorced/Separated

🞎 Other/Please specify: __________________________________

1. **What is the highest education you have completed?**

🞎 1^st^ cycle (4 years of education)

🞎 2^nd^ cycle (6 years of education)

🞎 3^rd^ cycle (9 years of education)

🞎 High school (12 years of education)

🞎 University / College

🞎 Other/Please specify: __________________________________

1. **What is your age (in years)?**

From 15 to 55:

1. **Is Portuguese your mother tongue?**

🞎 Yes 🞎 No

1. **Please specify your current pregnancy status.**

🞎 I am pregnant

🞎 I have given birth in the last 12 months.

1. (If you answered “I am pregnant”): **In which pregnancy week are you?**

From 1 to 44

1. (If yes to “I have recently given birth in the last 12 months”): **How old is your child?**

From 0 to 12 months

1. (If yes to “I have recently given birth in the last 12 months”): **Are you currently breastfeeding or have you breastfed your child?**

🞎 Yes 🞎 No

1. (If yes to “Are you currently breastfeeding or have you breastfed your child?): **What kind of breastfeeding?**

🞎 Exclusive with breastmilk

🞎 Partial with formula/milk

1. **Have you been pregnant before? (This also applies to pregnancy that ended in abortion, miscarriage or fetal death)**

🞎 Yes 🞎 No

1. **How many children do you have now?**

🞎 None 🞎 1 🞎 2 🞎 more than 2

INFORMATION ABOUT YOUR CURRENT OR LATEST PREGNANCY

1. **Was your pregnancy planned?**

🞎 Yes 🞎 No 🞎 No, but it was not completely unexpected

1. **Did you drink any alcohol after finding out that you were pregnant?**

🞎 Yes 🞎 No 🞎 Cannot remember

1. **Did you smoke after finding out you were pregnant?**

🞎 No 🞎 Yes, sometimes (2 to 3 times per week) 🞎 Yes, daily

1. **What was your work situation when you became pregnant?**

🞎 Student

🞎 Unemployed

🞎 Homemaker

🞎 Dependent worker

🞎 Independent worker

🞎 Other. Specify: ______________________________________.

- 1. Did that work situation change recently?

🞎 Yes 🞎 No

1. **When you got pregnant, were you working in the health sector (e.g., physician, nurse, pharmacist)?**

🞎 No

🞎 Yes. What was your job? _____________________________________.

CLINICAL HISTORY

1. **Do you or did you have any psychological problem or mental illness (for example, depression, anxiety) before, during or after your pregnancy?**

🞎 No

🞎 Yes

***[If NO, go to next section of the questionnaire]***

1. (If yes to question 19) **Check in the box the type of psychological problems you experienced and the period(s) in which you experienced that problem. Please choose all alternatives that apply to you.**

|  | More than 1 year before the pregnancy | 1 year or less before the pregnancy | During 1^st^ trimester of pregnancy | During 2^nd^ trimester of pregnancy | During 3^rd^ trimester of pregnancy | 0-6 months after birth | 7-12 months after birth |
| --- | --- | --- | --- | --- | --- | --- | --- |
| Depression | 󠄿 | 󠄿 | 󠄿 | 󠄿 | 󠄿 | 󠄿 | 󠄿 |
| Anxiety Disorder | 󠄿 | 󠄿 | 󠄿 | 󠄿 | 󠄿 | 󠄿 | 󠄿 |
| Obsessive Compulsive disorder | 󠄿 | 󠄿 | 󠄿 | 󠄿 | 󠄿 | 󠄿 | 󠄿 |
| Eating disorder (for example bulimia, anorexia, binge eating) | 󠄿 | 󠄿 | 󠄿 | 󠄿 | 󠄿 | 󠄿 | 󠄿 |
| Other mental illness | 󠄿 | 󠄿 | 󠄿 | 󠄿 | 󠄿 | 󠄿 | 󠄿 |

1. (If yes to question 19) **Did you recently receive or are you currently receiving any type of psychological treatment (e.g., psychotherapy)?**

🞎 Yes, I’m currently receiving psychological treatment;

🞎 Yes, I have received psychological treatment in the past, but not currently;

🞎 No, I do not receive or have not recently received any psychological treatment.

1. (If yes to question 21) **If yes, what kind? (for example individual psychotherapy, group therapy, counselling):**

**____________________________________________________________________________**

1. (If yes to question 21) **If yes, when? (check all option that apply to you)**

| More than 1 year before pregnancy | 1 year or less before pregnancy | During 1^st^ trimester of pregnancy | During 2^nd^ trimester of pregnancy | During 3^rd^ trimester of pregnancy | 0-6 months after birth | 7-12 months after birth |
| --- | --- | --- | --- | --- | --- | --- |
| 󠄿 | 󠄿 | 󠄿 | 󠄿 | 󠄿 | 󠄿 | 󠄿 |

MEDICATION USE

The next questions are about your use of antidepressant medication during pregnancy or the postpartum period.

1. **During your current pregnancy (or in your latest pregnancy), did you or are you taking antidepressant medication?**

🞎 No

🞎 Yes, I have previously taken and/or I am currently taking antidepressant medication

1. (“If yes to “I had a baby in the last 12 months”): **Did you take antidepressant medication for your psychological problem while you were breastfeeding?**

🞎 No

🞎 Yes, but the child received pumped milk when I took the medication(s)

🞎 Yes, irrespective of the type of antidepressant(s)

🞎 Yes, but I adapted the timing for breastfeeding according to the intake of the antidepressant

🞎 Cannot remember

🞎 Other, specify:_________________________________

**[If NO to the previous two questions, go to the next section of the questionnaire]**

1. (“If yes to questions 24 or 25”) **If you are currently taking or have previously taken antidepressant medication for your mental illness during the pregnancy or the postpartum periods, please select the relevant antidepressants from the list below and when you used them.**

|  | More than 6 months before pregnancy | 6 months or less before pregnancy | 1^st^ trimester of pregnancy | 2^nd^ trimester of pregnancy | 3^rd^ trimester of pregnancy | 0-6 months after birth | 7-12 months after birth |
| --- | --- | --- | --- | --- | --- | --- | --- |
| 🞎 Fluoxetine (incl. Prozac, etc) | 󠄿 | 󠄿 | 󠄿 | 󠄿 | 󠄿 | 󠄿 | 󠄿 |
| 🞎 Citalopram (incl. Citamil, Zitolex) | 󠄿 | 󠄿 | 󠄿 | 󠄿 | 󠄿 | 󠄿 | 󠄿 |
| 🞎 Escitalopram (incl. Cipralex) | 󠄿 | 󠄿 | 󠄿 | 󠄿 | 󠄿 | 󠄿 | 󠄿 |
| 🞎 Paroxetine (incl. Paxetil, Seroxat) | 󠄿 | 󠄿 | 󠄿 | 󠄿 | 󠄿 | 󠄿 | 󠄿 |
| 🞎 Sertraline (incl. Zoloft) | 󠄿 | 󠄿 | 󠄿 | 󠄿 | 󠄿 | 󠄿 | 󠄿 |
| 🞎 Fluvoxamine (incl. Dumyrox) | 󠄿 | 󠄿 | 󠄿 | 󠄿 | 󠄿 | 󠄿 | 󠄿 |
| 🞎 Venlafaxine (incl. Efexor, …) | 󠄿 | 󠄿 | 󠄿 | 󠄿 | 󠄿 | 󠄿 | 󠄿 |
| 🞎 Duloxetine (incl. Cymbalta) | 󠄿 | 󠄿 | 󠄿 | 󠄿 | 󠄿 | 󠄿 | 󠄿 |
| 🞎 Mirtazapine (incl. Remeron) | 󠄿 | 󠄿 | 󠄿 | 󠄿 | 󠄿 | 󠄿 | 󠄿 |
| 🞎 Reboxetine (incl. Edronax) | 󠄿 | 󠄿 | 󠄿 | 󠄿 | 󠄿 | 󠄿 | 󠄿 |
| 🞎 Mianserin (incl. Tolvon) | 󠄿 | 󠄿 | 󠄿 | 󠄿 | 󠄿 | 󠄿 | 󠄿 |
| 🞎 Amitriptyline (incl. ADT, Tryptizol) | 󠄿 | 󠄿 | 󠄿 | 󠄿 | 󠄿 | 󠄿 | 󠄿 |
| 🞎Clomipramine (incl. Anafranil) | 󠄿 | 󠄿 | 󠄿 | 󠄿 | 󠄿 | 󠄿 | 󠄿 |
| 🞎 Trimipramine (incl. Surmontil) | 󠄿 | 󠄿 | 󠄿 | 󠄿 | 󠄿 | 󠄿 | 󠄿 |
| 🞎 Nortriptyline (incl. Norterol) | 󠄿 | 󠄿 | 󠄿 | 󠄿 | 󠄿 | 󠄿 | 󠄿 |
| 🞎 Doxepine  (incl. Quitaxon) | 󠄿 | 󠄿 | 󠄿 | 󠄿 | 󠄿 | 󠄿 | 󠄿 |

1. (“If yes to question 24”) **Have you purposely stopped taking your prescribed antidepressant(s) during pregnancy?**

🞎 Yes 🞎 No 🞎 Cannot remember

1. (If yes to question 27) **Who recommended you to avoid antidepressant in pregnancy?**

🞎 Physician

🞎 Nurse

🞎 Midwife

🞎 Pharmacy personnel

🞎 Family/Friends

🞎 Internet

🞎 Nobody, my own initiative

1. (“If yes to question 24”) **Was the dose of your prescribed antidepressant changed during pregnancy?**

🞎 No

🞎 Yes, increased

🞎 Yes, reduced

🞎 Yes, I stopped taking the medication

1. **If you are taking or have been taking other medications than antidepressants for your mental illness during the period of pregnancy, please choose relevant medications from the list below, and when you were using them.**

|  | 6 months or less before pregnancy | 1^st^ trimester | 2^nd^ trimester | 3^rd^ trimester | After birth |
| --- | --- | --- | --- | --- | --- |
| Paracetamol (for example, Ben-u-ron, Panadol) |  |  |  |  |  |
| Opioid analgesics (for exemple, Tramadol) |  |  |  |  |  |
| Lithium (Priadel) |  |  |  |  |  |
| Antipsychotics (for example, Zyprexa, Seroquel) |  |  |  |  |  |
| Anxiolytics (for example, Valium, Xanax, Olcadil, Ativan) |  |  |  |  |  |
| Sleeping medications (for example, Zolpidem, Triticum) |  |  |  |  |  |

YOUR DECISION-MAKING ABOUT ANTIDEPRESSANT TREATMENT

[VERSION FOR THOSE WHO TOOK ANTIDEPRESSANT MEDICATION OR RECEIVED PSYCHOLOGICAL TREATMENT DURING PREGNANCY OR THE POSTPARTUM]

The next questions are about your decision-making difficulties related to use of antidepressants or other treatment options (e.g., psychological treatment) in the period around pregnancy and the postpartum. There are no right or wrong answers. We are only interested in your personal views.

## **Which treatment option was your preferred during pregnancy or the postpartum?**

🞎 Pharmacological treatment with antidepressants

🞎 Non-pharmacological treatment (e.g., psicotherapy)

🞎 Combined non-pharmacological with antidepressants & therapy

🞎 No treatment

🞎 Unsure

1. **Was your preferred option selected?**

🞎 Yes

🞎 No

1. **On a scale from 0 (not taken into account) to 10 (totally taken into account), how much do you think your opinion was taken into account in the selection of the treatment option?**

0 1 2 3 4 5 6 7 8 9 10

1. **Who took part in the decision-making (you can select all options that apply to you)?**

🞎 Me

🞎 My doctor

🞎 My partner

🞎 Someone else. Who? ____________________________

1. Considering the decision-making process that you had to go through to select your treatment option, please answer the next questions:

|  |  | Strongly agree [0] | Agree [1] | Neither agree nor disagree  [2] | Disagree [3] | Strongly disagree [4] |
| --- | --- | --- | --- | --- | --- | --- |
| 1. | I was aware of the options that were available for me. | 󠄿 | 󠄿 | 󠄿 | 󠄿 | 󠄿 |
| 2. | I knew the benefits of each option. | 󠄿 | 󠄿 | 󠄿 | 󠄿 | 󠄿 |
| 3. | I knew the risks and side effects of each option. | 󠄿 | 󠄿 | 󠄿 | 󠄿 | 󠄿 |
| 4. | I was aware of the benefits that mattered most to me. | 󠄿 | 󠄿 | 󠄿 | 󠄿 | 󠄿 |
| 5. | I was aware of the risks and side effects that mattered the most to me. | 󠄿 | 󠄿 | 󠄿 | 󠄿 | 󠄿 |
| 6. | I was aware of what weighed more for me (benefits versus risks and side effects). | 󠄿 | 󠄿 | 󠄿 | 󠄿 | 󠄿 |
| 7. | I had enough support from others to make a choice. | 󠄿 | 󠄿 | 󠄿 | 󠄿 | 󠄿 |
| 8. | My choice was not pressured by other people. | 󠄿 | 󠄿 | 󠄿 | 󠄿 | 󠄿 |
| 9. | I had enough advice to make my choice. | 󠄿 | 󠄿 | 󠄿 | 󠄿 | 󠄿 |
| 10. | I was aware of what was the best choice for me. | 󠄿 | 󠄿 | 󠄿 | 󠄿 | 󠄿 |
| 11. | I was sure about what to choose. | 󠄿 | 󠄿 | 󠄿 | 󠄿 | 󠄿 |
| 12. | For me, this was an easy decision to make. | 󠄿 | 󠄿 | 󠄿 | 󠄿 | 󠄿 |
| 13. | I feel like I made an informed choice. | 󠄿 | 󠄿 | 󠄿 | 󠄿 | 󠄿 |
| 14. | My decision reflects what is important to me. | 󠄿 | 󠄿 | 󠄿 | 󠄿 | 󠄿 |
| 15. | I hope to keep my decision. | 󠄿 | 󠄿 | 󠄿 | 󠄿 | 󠄿 |
| 16. | I’m happy with my decision. | 󠄿 | 󠄿 | 󠄿 | 󠄿 | 󠄿 |

1. **The next questions are about your relationship with your doctor and your partner in the decision-making process, as well as your attitudes towards mental illness. Please indicate the degree to which you agree with each sentence. There are no right or wrong answers. We are only interested in your personal views.**

a) **Your relationship with your doctor**

|  |  | Strongly disagree | Disagree | Agree | Strongly agree |
| --- | --- | --- | --- | --- | --- |
| 1. | My doctor has explained properly about antidepressants, their actions and side effects. | 󠄿 | 󠄿 | 󠄿 | 󠄿 |
| 2. | My doctor adequately explained to me what psychological interventions are possible, how they work, and their effectiveness. |  |  |  |  |
| 3. | My doctor shows sufficient consideration for my opinions and feelings regarding treatment with antidepressants. | 󠄿 | 󠄿 | 󠄿 | 󠄿 |
| 4. | My doctor shows sufficient consideration for my opinions and feelings about non-pharmacological treatment options (e.g., psychotherapy). |  |  |  |  |
| 5. | My doctor fully understands my condition. | 󠄿 | 󠄿 | 󠄿 | 󠄿 |

b) **Your relationship with your partner**

|  |  | SD | D | A | SA |
| --- | --- | --- | --- | --- | --- |
| 1. | My partner agrees that antidepressants are a suitable treatment for my condition | 󠄿 | 󠄿 | 󠄿 | 󠄿 |
| 2. | My partner agrees that non-pharmological treatment options (e.g., psychotherapy) are a suitable treatment for my condition. |  |  |  |  |
| 3. | My partner fully understands my condition. |  |  |  |  |
| 4. | My partner does not want to involved in the decisions about my treatment. |  |  |  |  |

c) **Your attitudes regarding mental illness**

0-Disagree; 1-Partly disagree; 2-Indecided; 3-Partly agree; 4-Agree

|  | 0 | 1 | 2 | 3 | 4 |
| --- | --- | --- | --- | --- | --- |
| 1. I would not want my partner to know about my psychological problems. |  |  |  |  |  |
| 1. I feel ashamed for my psychological problems. |  |  |  |  |  |
| 1. Important people in my life think less of me since they found out that I was experiencing psychological problems. |  |  |  |  |  |
| 1. I feel uncomfortable seeking professional help for psychological problems because people in my social circles might find out about it |  |  |  |  |  |
| 1. Having been diagnosed with a mental disorder is a blot in my life. |  |  |  |  |  |
| 1. I feel uneasy going to a mental health professional because of what some people might think. |  |  |  |  |  |
| 1. I do not feel that I have to “cover up” that I am receiving treatment for psychological problems. |  |  |  |  |  |
| 1. I would be embarrassed if my neighbor saw me going into the office of a professional who deals with psychological problems |  |  |  |  |  |

YOUR DECISION-MAKING ABOUT ANTIDEPRESSANT TREATMENT

[VERSION FOR THOSE WHO DID NOT TOOK ANTIDEPRESSANT MEDICATION OR RECEIVED PSYCHOLOGICAL TREATMENT DURING PREGNANCY OR THE POSTPARTUM]

In the next questions, we ask you to think about the possibility of having a psychological problem during pregnancy or the postpartum period and that you reflect about your decision-making process related to the treatment of that problem. There are no right or wrong answers. We are only interested in your personal views.

## **If you had a psychological problem during pregnancy or the postpartum, which treatment option would you prefer?**

🞎 Pharmacological treatment with antidepressants

🞎 Non-pharmacological treatment (e.g., psicotherapy)

🞎 Combined non-pharmacological with antidepressants & therapy

🞎 No treatment

🞎 Unsure

1. **If you had a psychological problem during pregnancy or the postpartum, on a scale from 0 (not taken into account) to 10 (totally taken into account), how much do you think your opinion would be taken into account in the selection of the treatment option?**

0 1 2 3 4 5 6 7 8 9 10

1. **If you had a psychological problem during pregnancy or the postpartum, who would take part in the decision-making (you can select all options that apply to you)?**

🞎 Me

🞎 My doctor

🞎 My partner

🞎 Someone else. Who? ____________________________

## **If you had a psychological problem before your pregnancy and you were taking antidepressants, what would be your preference in regards to treatment with antidepressants during pregnancy?**

🞎 Continuing treatment with the same antidepressant(s)

🞎 Changing to another antidepressant

🞎 Stop taking the antidepressant

🞎 Reduce the dose of the antidepressant

🞎 No preference

🞎 Other, specify: _________________________________

1. Considering the possibility of having a psychological problem during pregnancy or in the postpartum period, we ask you to reflect about the decision you would take on available treatments. Keeping that in mind, please answer the next questions:

|  |  | Strongly agree [0] | Agree [1] | Neither agree nor disagree  [2] | Disagree [3] | Strongly disagree [4] |
| --- | --- | --- | --- | --- | --- | --- |
| 1. | I would be aware of the options that were available for me. | 󠄿 | 󠄿 | 󠄿 | 󠄿 | 󠄿 |
| 2. | I would know the benefits of each option. | 󠄿 | 󠄿 | 󠄿 | 󠄿 | 󠄿 |
| 3. | I would know the risks and side effects of each option. | 󠄿 | 󠄿 | 󠄿 | 󠄿 | 󠄿 |
| 4. | I would be aware of the benefits that matter most to me. | 󠄿 | 󠄿 | 󠄿 | 󠄿 | 󠄿 |
| 5. | I would be aware of the risks and side effects that mattered the most to me. | 󠄿 | 󠄿 | 󠄿 | 󠄿 | 󠄿 |
| 6. | I would be aware of what weighed more for me (benefits versus risks and side effects). | 󠄿 | 󠄿 | 󠄿 | 󠄿 | 󠄿 |
| 7. | I would have enough support from others to make a choice. | 󠄿 | 󠄿 | 󠄿 | 󠄿 | 󠄿 |
| 8. | My choice would not be pressured by other people. | 󠄿 | 󠄿 | 󠄿 | 󠄿 | 󠄿 |
| 9. | I would have enough advice to make my choice. | 󠄿 | 󠄿 | 󠄿 | 󠄿 | 󠄿 |
| 10. | I would be aware of what was the best choice for me. | 󠄿 | 󠄿 | 󠄿 | 󠄿 | 󠄿 |
| 11. | I would be sure about what to choose. | 󠄿 | 󠄿 | 󠄿 | 󠄿 | 󠄿 |
| 12. | For me, this would be an easy decision to make. | 󠄿 | 󠄿 | 󠄿 | 󠄿 | 󠄿 |
| 13. | I would feel like I made an informed choice. | 󠄿 | 󠄿 | 󠄿 | 󠄿 | 󠄿 |
| 14. | My decision would reflect what is important to me. | 󠄿 | 󠄿 | 󠄿 | 󠄿 | 󠄿 |
| 15. | I would hope to keep my decision. | 󠄿 | 󠄿 | 󠄿 | 󠄿 | 󠄿 |
| 16. | I would be happy with my decision. | 󠄿 | 󠄿 | 󠄿 | 󠄿 | 󠄿 |

1. **Once again, we ask you to think about the possibility of having a psychological problem during pregnancy or the postpartum period and that you reflect about the decision of your treatment options. The next questions are about the possible relationship with your doctor and your partner in the decision-making process, as well as your attitudes towards mental illness. Please indicate the degree to which you agree with each sentence. There are no right or wrong answers. We are only interested in your personal views.**

a) **Your relationship with your doctor**

|  |  | Strongly disagree | Disagree | Agree | Strongly agree |
| --- | --- | --- | --- | --- | --- |
| 1. | My doctor would explain to me properly about antidepressants, their actions and side effects. | 󠄿 | 󠄿 | 󠄿 | 󠄿 |
| 2. | My doctor would adequately explain to me what psychological interventions are possible, how they work, and their effectiveness. |  |  |  |  |
| 3. | My doctor would show sufficient consideration for my opinions and feelings regarding treatment with antidepressants. | 󠄿 | 󠄿 | 󠄿 | 󠄿 |
| 4. | My doctor would show sufficient consideration for my opinions and feelings about non-pharmacological treatment options (e.g., psychotherapy). |  |  |  |  |
| 5. | My doctor would fully understand my condition. | 󠄿 | 󠄿 | 󠄿 | 󠄿 |

b) **Your relationship with your partner**

|  |  | SD | D | A | SA |
| --- | --- | --- | --- | --- | --- |
| 1. | My partner would agree that antidepressants are a suitable treatment for my condition | 󠄿 | 󠄿 | 󠄿 | 󠄿 |
| 2. | My partner would agree that non-pharmological treatment options (e.g., psychotherapy) are a suitable treatment for my condition. |  |  |  |  |
| 3. | My partner would fully understand my condition. |  |  |  |  |
| 4. | My partner would not want to involved in the decisions about my treatment. |  |  |  |  |

c) **Your attitudes regarding mental illness**

0-Disagree; 1-Partly disagree; 2-Indecided; 3-Partly agree; 4-Agree

|  | 0 | 1 | 2 | 3 | 4 |
| --- | --- | --- | --- | --- | --- |
| 1. If I had a psychological problem, I would not want my partner to know about it. |  |  |  |  |  |
| 1. Having a mental illness carries a burden of shame. |  |  |  |  |  |
| 1. Important people in my life would think less of me if they found out that I was experiencing psychological problems. |  |  |  |  |  |
| 1. I would feel uncomfortable seeking professional help for psychological problems because people in my social circles might find out about it |  |  |  |  |  |
| 1. Being diagnosed with a mental disorder is a blot in my life. |  |  |  |  |  |
| 1. I would feel uneasy going to a mental health professional because of what some people might think. |  |  |  |  |  |
| 1. If I had received treatment for psychological problems, I would not feel that I should hide it. |  |  |  |  |  |
| 1. I would be embarrassed if my neighbor saw me going into the office of a professional who deals with psychological problems |  |  |  |  |  |

YOUR PERCEPTION OF RISK DURING PREGNANCY AND WHILE BREASTFEEDING

1. **Do you consider that antidepressants can be used safely in all stages of pregnancy? (You can select more than one answer)**

🞎 No

🞎 There should be professional, personalized counseling to help the woman decide whether or not to opt for pharmacological treatment

🞎 The use should be discontinued because it is harmful to the fetus/baby

🞎 The use should not be discontinued, because it can be detrimental to the mother's mental health

🞎 No opinion

🞎 Other, specify:_________________________________

1. **Below is a list with various medications, conditions, food and other substances. Please indicate how harmful you think they are during pregnancy and lactation on a scale from 0 to 10, where 0 corresponds to ‘not harmful’ and 10 to ‘very harmful’. With the word “harmful”, we mean in relation to child longer-term development (for example autism, motor or language development, ADHD). If you do not know or have not heard of the condition, medication, food, or substance, check the "Unknown" box.**

|  | Unknown | 0 | 1 | 2 | 3 | 4 | 5 | 6 | 7 | 8 | 9 | 10 |
| --- | --- | --- | --- | --- | --- | --- | --- | --- | --- | --- | --- | --- |
| ***How dangerous are these (conditions, medications, foods or substances) during pregnancy for your child development?*** | ◦ | ◦ | ◦ | ◦ | ◦ | ◦ | ◦ | ◦ | ◦ | ◦ | ◦ | ◦ |
| Maternal psychiatric disorder |  |  |  |  |  |  |  |  |  |  |  |  |
| Antidepressants | ◦ | ◦ | ◦ | ◦ | ◦ | ◦ | ◦ | ◦ | ◦ | ◦ | ◦ | ◦ |
| Antipsychotics | ◦ | ◦ | ◦ | ◦ | ◦ | ◦ | ◦ | ◦ | ◦ | ◦ | ◦ | ◦ |
| Anxiolytic benzodiazepines and sleeping drugs | ◦ | ◦ | ◦ | ◦ | ◦ | ◦ | ◦ | ◦ | ◦ | ◦ | ◦ | ◦ |
| Antiepileptics (e.g., valproate) |  |  |  |  |  |  |  |  |  |  |  |  |
| Cranberry | ◦ | ◦ | ◦ | ◦ | ◦ | ◦ | ◦ | ◦ | ◦ | ◦ | ◦ | ◦ |
| Alcohol (e.g. wine, beer, spirits) | ◦ | ◦ | ◦ | ◦ | ◦ | ◦ | ◦ | ◦ | ◦ | ◦ | ◦ | ◦ |
| *Tobaco* |  |  |  |  |  |  |  |  |  |  |  |  |
| *Soft drugs (e.g., marijuana)* |  |  |  |  |  |  |  |  |  |  |  |  |
| ***How dangerous are these while breastfeeding for your child development?*** | ◦ | ◦ | ◦ | ◦ | ◦ | ◦ | ◦ | ◦ | ◦ | ◦ | ◦ | ◦ |
| Maternal psychiatric disorder |  |  |  |  |  |  |  |  |  |  |  |  |
| Antidepressants | ◦ | ◦ | ◦ | ◦ | ◦ | ◦ | ◦ | ◦ | ◦ | ◦ | ◦ | ◦ |
| Antipsychotics | ◦ | ◦ | ◦ | ◦ | ◦ | ◦ | ◦ | ◦ | ◦ | ◦ | ◦ | ◦ |
| Anxiolytic benzodiazepines and sleeping drugs | ◦ | ◦ | ◦ | ◦ | ◦ | ◦ | ◦ | ◦ | ◦ | ◦ | ◦ | ◦ |
| Antiepileptics (e.g., valproate) |  |  |  |  |  |  |  |  |  |  |  |  |
| Cranberry | ◦ | ◦ | ◦ | ◦ | ◦ | ◦ | ◦ | ◦ | ◦ | ◦ | ◦ | ◦ |
| Alcohol (e.g. wine, beer, spirits) | ◦ | ◦ | ◦ | ◦ | ◦ | ◦ | ◦ | ◦ | ◦ | ◦ | ◦ | ◦ |

**Thank you for your help!**
